# Acute aerobic exercise‐conditioned serum reduces colon cancer cell proliferation in vitro through interleukin‐6‐induced regulation of DNA damage

**DOI:** 10.1002/ijc.33982

**Published:** 2022-03-05

**Authors:** Samuel T. Orange, Alastair R. Jordan, Adam Odell, Owen Kavanagh, Kirsty M. Hicks, Tristan Eaglen, Stephen Todryk, John M. Saxton

**Affiliations:** ^1^ School of Biomedical, Nutritional and Sport Sciences, Faculty of Medical Sciences Newcastle University Newcastle upon Tyne UK; ^2^ Newcastle University Centre for Cancer, Newcastle University Newcastle upon Tyne UK; ^3^ School of Science, Technology and Health York St John University York UK; ^4^ Department of Sport, Exercise and Rehabilitation, Faculty of Health and Life Sciences Northumbria University Newcastle upon Tyne UK; ^5^ Department of Applied Sciences, Faculty of Health and Life Sciences Northumbria University Newcastle upon Tyne UK; ^6^ Department of Sport, Health and Exercise Science, Faculty of Health Sciences University of Hull Hull UK

**Keywords:** acute exercise, cancer therapy, colon cancer, exercise‐oncology, physical activity

## Abstract

Epidemiological evidence shows that regular physical activity is associated with reduced risk of primary and recurrent colon cancer. However, the underlying mechanisms of action are poorly understood. We evaluated the effects of stimulating a human colon cancer cell line (LoVo) with human serum collected before and after an acute exercise bout vs nonexercise control serum on cancer cell proliferation. We also measured exercise‐induced changes in serum cytokines and intracellular protein expression to explore potential biological mechanisms. Blood samples were collected from 16 men with lifestyle risk factors for colon cancer (age ≥50 years; body mass index ≥25 kg/m^2^; physically inactive) before and immediately after an acute bout of moderate‐intensity aerobic interval exercise (6 × 5 minutes intervals at 60% heart rate reserve) and a nonexercise control condition. Stimulating LoVo cells with serum obtained immediately after exercise reduced cancer cell proliferation compared to control (−5.7%; *P* = .002). This was accompanied by a decrease in LoVo cell γ‐H2AX expression (−24.6%; *P* = .029), indicating a reduction in DNA damage. Acute exercise also increased serum IL‐6 (24.6%, *P* = .002). Furthermore, stimulating LoVo cells with recombinant IL‐6 reduced γ‐H2AX expression (*β* = −22.7%; *P* < .001) and cell proliferation (*β* = −5.3%; *P* < .001) in a linear dose‐dependent manner, mimicking the effect of exercise. These findings suggest that the systemic responses to acute aerobic exercise inhibit colon cancer cell proliferation in vitro, and this may be driven by IL‐6‐induced regulation of DNA damage and repair. This mechanism of action may partly underlie epidemiological associations linking regular physical activity with reduced colon cancer risk.

## Graphical abstract

What's new?

While regular physical activity can lower colon cancer risk, the mechanisms underlying this association remain unknown. Here, the authors investigated mechanisms linking exercise and cell proliferation in human colon cancer cells exposed to human serum collected before and after acute exercise. Compared to nonexercise control serum, serum obtained after exercise was found to reduce cancer cell proliferation. This effect was accompanied by decreased expression of the DNA damage marker γ‐H2AX. Reductions in growth and DNA damage were linked to exercise‐induced increases in serum IL‐6. The newly identified mechanism may partly underlie associations between physical activity and reduced colon cancer risk.

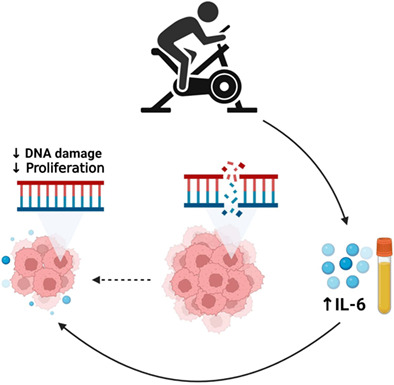

**Abbreviations**
95% CI: 95% confidence interval
BMI: body mass index
BSA: bovine serum albumin
DMEM: Dulbecco's modified Eagle's medium
DSB: double‐strand break
EV: extracellular vesicle
FBS: fetal bovine serum
HRP: horseradish peroxidase
IL‐10: interleukin‐10
IL‐6: interleukin‐6
IL‐8: interleukin‐8
IQR: interquartile range
PBS: phosphate‐buffered saline
RPE: rating of perceived exertion
SDS‐PAGE: sodium dodecyl sulphate‐polyacrylamide gel electrophoresis
STR: short‐tandem repeat
TNF‐α: tumour necrosis factor‐alpha

## 1 INTRODUCTION

There is strong epidemiological evidence that regular physical activity protects against colon cancer.^1^, ^2^, ^3^ Current estimates suggest that achieving the highest vs lowest level of physical activity reduces the relative risk of developing colon cancer by 12% to 28%.^1^ Physical activity after a colon cancer diagnosis is also associated with a decreased risk of cancer‐specific mortality^2^ and recurrence.^4^

The biological mechanisms underlying how physical activity reduces colon cancer risk have mainly been attributed to decreased adiposity and associated reductions in circulating insulin and proinflammatory cytokines.^5^, ^6^ However, the epidemiological evidence, including both observational and Mendelian randomisation studies, demonstrates that physical activity is inversely related to colon cancer risk independent of adiposity.^7^, ^8^, ^9^, ^10^ Murine studies also show that physical activity alone, without dietary modification or changes in body weight, reduces tumour progression.^11^, ^12^ This indicates that physical activity might reduce colon cancer risk—at least partly—through biological mechanisms other than body fat‐associated pathways.

Exercise is a subcomponent of physical activity encompassing planned, structured activities purposefully carried out to improve physical fitness.^13^ During a bout of exercise, skeletal muscle and other secretory organs release bioactive molecules (proteins, nucleic acids, metabolites) into the systemic circulation.^14^ These molecules can elicit biological effects on distant cells via endocrine‐like signalling and are thought to mediate some of the multisystemic benefits of exercise.^14^

Early preclinical evidence shows that the systemic responses to exercise can regulate cancer cell proliferation in vitro.^15^, ^16^ Indeed, our recent meta‐analysis demonstrated that stimulating a range of cancer cell lines with human serum obtained immediately after exercise reduces cell proliferation by ≈9%.^17^ The transient modulation of humoral factors during acute exercise may represent a novel mechanism underlying the association between regular physical activity and reduced colon cancer risk. However, only one study included in the meta‐analysis used a cell line model of colorectal cancer, comprising an uncontrolled, prepost design (n = 10 colorectal cancer survivors).^18^ The inclusion of a nonexercise control experiment is needed to account for biological variation and regression to the mean effects.^19^ Previously we have also highlighted concerns about the overall quality of evidence,^15^, ^17^ warranting the conduct of well‐controlled studies to strengthen the evidence‐base.

An improved understanding of the precise signalling molecules and pathways driving the growth‐inhibitory effects of exercise could help inform guidelines for the optimal exercise dose needed for cancer prevention. It could also increase the likelihood of physical activity being integrated into standard cancer preventive care as a therapeutic intervention. Therefore, our study assessed the effects of stimulating a colon cancer cell line with human serum collected before and after an acute exercise bout vs nonexercise control serum on colon cancer cell proliferation. We also quantified exercise‐induced changes in serum cytokines and intracellular protein expression to explore potential molecular mechanisms of action.

## 2 METHODS

### 2.1 Participants

We recruited males who had lifestyle risk factors for colon cancer between August 2019 and February 2020. Inclusion criteria were: age ≥50 years, body mass index (BMI) ≥25 kg/m^2^ or waist circumference ≥94 cm, and not engaged in ≥30 minutes of moderate to vigorous‐intensity physical activity on ≥3 days·wk^−1^ for the last 3 months. Main exclusion criteria were: signs/symptoms of cardiovascular, metabolic or renal disease, hypertension (≥160/≥90 mm Hg), previous stroke, taking beta‐adrenergic blocking agents, previous treatment for malignancy, respiratory disease with peak respiratory flow <300 L/min, or any musculoskeletal, neurological, or rheumatoid condition that could be exasperated due to exercise.

### 2.2 Study design

Our study used a two‐site, prospective, randomised, controlled, crossover design. Participants completed an acute bout of moderate‐intensity aerobic interval exercise and a nonexercise control experiment in a randomised, counterbalanced order, separated by 2 to 7 days (median: 6 days). The randomisation sequence was stratified by site and generated in block sizes of four by an independent researcher using online randomisation software. The order was concealed from the research team using opaque, sealed envelopes until eligibility was confirmed. Before visiting the laboratory, participants were instructed to eat the same meal 2 to 5 hours prior, not to engage in moderate‐ to vigorous‐intensity physical activity or consume alcohol for ≥24 hours, avoid caffeine intake for ≥12 hours, and to arrive fully hydrated.

### 2.3 Acute exercise bout

The moderate‐intensity aerobic interval exercise was performed on a cycle ergometer (Lode Excalibur sport, Groningen, Netherlands) under the supervision of research staff in the exercise science facilities at Northumbria University and York St John University (both UK). Following a 10 minute warm‐up that involved pedalling against a light resistance (60 W), participants completed 6 × 5 minute intervals at 60% heart rate reserve, separated by 2.5 minutes of active recovery (60 W). Heart rate reserve was determined as the difference between an individual's resting and estimated maximum heart rate (220 minus age). We employed an interval exercise protocol rather than continuous exercise to ensure all participants were able to complete a total of 30 minutes of moderate‐intensity exercise without reaching the limit of tolerance. A pedal cadence of 60 rev·min^−1^ was maintained throughout. Heart rate was monitored continuously, rating of perceived exertion (RPE) was collected in the final minute of each 5 minute interval using the 6‐20 Borg scale,^20^ and blood pressure was measured once during each active recovery period. The load was reduced by ≈10% if heart rate reserve increased to >65% or RPE was >14.

### 2.4 Nonexercise control

The control experiment intended to control for the natural deviation of serum analytes, and thus serum‐stimulated cell proliferation, in the absence of exercise. The experiment involved 60 minutes of quiet, seated rest, conducted at the same time of day as the acute exercise bout (±1 hour) to control for diurnal variation.

### 2.5 Serum collection

Blood samples were drawn before the warm‐up and immediately after completing the acute exercise bout, and at the same time‐points before and after the control experiment. Each ≈20 mL blood sample was drawn from an antecubital vein and collected in 10 mL Vacutainer serum tubes (BD, New Jersey). Samples were allowed to clot at room temperature for 60 minutes, centrifuged at 1000*g* for 20 minutes, apportioned into 0.5 to 1 mL aliquots, and cryopreserved at −80°C for later analysis.

### 2.6 Cell line

A human colon cancer cell line (LoVo, RRID:CVCL_0399) was purchased from Sigma‐Aldrich (Dorset, UK) and cultured in Dulbecco's modified Eagle's medium (DMEM) supplemented with 4500 mg/L glucose, 10% fetal bovine serum (FBS), 1% glutamine and 1% penicillin‐streptomycin. We chose to use the LoVo cell line because it harbours *APC* and *KRAS* mutations but is wildtype for *TP53*,^21^ which are genetic features associated with the early stages of cancer development.^22^ LoVo cells also have the genomic instability phenotype microsatellite instability.^21^ Thus, the LoVo cell line serves as a useful model to study the potential role of exercise in modulating early colorectal carcinogenesis through the oncogene‐induced DNA damage model for cancer development.^23^ Cells were thawed and passaged 4 to 8 times at ≈70% confluence before being used for experiments, and were maintained at 37°C in a humidified atmosphere of 5% CO_2_. The cell line was authenticated using short‐tandem repeat (STR) profiling within the previous year (NorthGene, Newcastle upon Tyne, UK). All experiments were performed with mycoplasma‐free cells.

### 2.7 Outcomes

The prespecified primary outcome was mean difference in LoVo cell proliferation between exercise and control conditions. Secondary outcomes, used to explore potential molecular mechanisms of action, included exercise‐induced changes in serum cytokine concentration and LoVo cell protein expression.

#### 2.7.1 Cell proliferation

Viability of the LoVo cells was assessed via quantification of the fluorescent signal by the resazurin assay (Sigma‐Aldrich). Following trypsinisation, cells were counted with a haemocytometer and seeded at 1 × 10^4^ cells·well^−1^ in quintuplets within opaque, clear‐bottom 96‐well plates (Greiner Bio One Ltd, Stonehouse, UK). Cells were not seeded in outer wells to avoid the potential influence of unequal evaporation on differences in fluorescent signal across the plate. Serum samples from each individual were used on the same 96‐well plate to negate interplate variability. Cells were initially seeded in 100 μL of their normal growth medium for 24 hours to allow attachment to the bottom of the plate. The normal growth medium was then aspirated and replaced with 100 μL of DMEM containing 1000 mg/L glucose, 1% glutamine, and 10% serum from individual participants instead of FBS. Stimulated cells were incubated for 48 hours. Resazurin dye was then added to each well at a final concentration of 0.02%, mixed via orbital shaking, and incubated for a further 4 hours. Fluorescence was measured using a microplate reader at an excitation of 540 nm and emission of 590 nm. Background fluorescence was subtracted from each well and then values were normalised to fluorescence of control cells grown in 10% FBS, with the mean value five replicate wells used for analysis. LoVo cell proliferation following direct IL‐6 stimulation was assessed by standard MTT assay after 48 hours of incubation.

#### 2.7.2 Serum cytokines

Serum concentrations of seven cytokines/myokines were quantified using bead‐based multiplex immunoassays (Milliplex, Merck Millipore, Burlington). Interleukin 6 (IL‐6), IL‐8, IL‐10, and tumour necrosis factor‐alpha (TNF‐α) were assessed by a human cytokine 4‐plex panel (HYCTA‐60 K), and irisin, osteonectin, and oncostatin M were quantified by a human myokine 3‐plex panel (HMYOMAG‐56 K). All samples were run in duplicate and the assay was performed according to the manufacturer's instructions. Plates were read on the Luminex MAGPIX system (Merck Millipore). Median intraassay coefficient of variations for each analyte ranged from 4.1% to 10.7%.

#### 2.7.3 Western blot

LoVo cells are *KRAS* mutant but wildtype for several other colon cancer critical genes (eg, TP53, BRAF, PTEN).^21^ Thus, we mainly focused on components of the MAPK/ERK pathway, as well as the mTOR pathway, because of their relevance to colon cancer and potential to be modified by acute exercise. Phospho‐specific antibodies MEK1/2 (#9154), ERK1/2 (#4370), CREB1 (#9198), ATF1 (#9198), RSK90 (#9344), NF‐κB (#3033), Akt (#4060), mTOR (#5536), histone H2AX (γ‐H2AX; #9718), β‐actin (#8457) and α‐Tubulin (#2144) were purchased from Cell Signalling Technology (CST; Beverly, Massachusetts). Briefly, LoVo cells were trypsinised and seeded at 2x10^5^ cells.well^−1^ in 12‐well plates. Upon reaching 80% to 90% confluence, cells were gently washed once in serum‐free DMEM before incubation in serum‐free DMEM supplemented with 0.2% bovine serum albumin (BSA) for 2 hours. Serum‐starved LoVo cells were then treated with 10% participant serum samples for either 20 or 60 minutes before washing 3 times in cold phosphate‐buffered saline (PBS) and lysis in 2% sodium dodecyl sulphate (SDS) lysis buffer supplemented with complete protease inhibitors (Roche, Mannheim, Germany). Protein concentration was determined by micro BCA assay according to the manufacturer's instructions (ThermoFisher, Cramlington, UK). Thirty to thirty‐five micrograms of total protein was separated on 5% to 17% gradient SDS‐polyacrylamide gel electrophoresis (SDS‐PAGE) followed by Western blotting. Primary antibodies (1:2000 dilution) were prepared in TBST containing 0.5% BSA and 0.01% sodium azide and incubated overnight. Antirabbit (#7074) and antimouse (#7076) horseradish peroxidase (HRP) conjugated secondary antibodies were prepared in TBST at 1:5000 dilution and incubated for 1 hour. Western blots were detected using Clarity ELC substrate (Bio‐Rad Laboratories, Hertfordshire, UK) and captured on an iBright CL1000 imaging system (ThermoFisher).

### 2.8 Sample size

We used Monte Carlo simulations to estimate the sample size required to achieve at least 80% statistical power to detect a difference in the primary outcome (LoVo cell proliferation), assuming certain population parameters. Based on previous research,^18^ we generated a random‐sampled, normally‐distributed data set assuming cell viability of 54 ± 3.5% at baseline, and a reduction in cell viability of 3 ± 3.5% following stimulation with exercise‐conditioned serum. We then fit a linear mixed model to the data set and calculated power as the percentage of statistically significant *P*‐values (*P* ≤ .05) based on 1 × 10^4^ simulations.^24^ A total of 15 participants were required to achieve approximately 89% power. Simulations were performed in R version 3.6.1 (R Foundation for Statistical Computing, Vienna, Austria) and reproducible code is available on the Open Science Framework (OSF) repository.^25^

### 2.9 Statistical analysis

Difference in serum‐stimulated cell proliferation between exercise and control conditions was assessed with a linear mixed model. Change from baseline (delta) was the dependent variable, baseline values were entered as a covariate, condition was a fixed effect with two levels (exercise and control), and participants were a random factor with individual slopes.^26^ Exercise‐induced changes in serum markers and intracellular protein expression were assessed a using linear mixed model with time‐point as a fixed effect (pre and post) and participants as a random factor. Models were fit using the maximum likelihood method. Normality of model residuals were assessed via visual inspection of histograms and Q‐Q plots. Positively skewed data were analysed with a generalised linear mixed model specifying a gamma distribution and log link function,^27^ with goodness of fit compared between models using the Bayesian information criterion. When multiple comparisons were made in a “family” of tests, we applied a Bonferroni correction to control the familywise error rate. Linear trend analysis was used to assess the dose‐response effect of IL‐6 on LoVo cell proliferation and γ‐H2AX. Statistical significance was set at *P* < .05. Analyses were performed in R. Data and code are available on OSF.^25^

## 3 RESULTS

Sixteen participants completed the exercise and control experiments (Table 1). Participant flow through the study is presented in Figure S1. All participants reported that they consumed the same meal 2 to 5 hours prior to laboratory visits and avoided physical activity and alcohol intake for ≥24 hours and caffeine intake for ≥12 hours prior.

**TABLE 1:** Participant characteristics (n = 16)

Characteristic	Mean ± SD or number (%)
Age (y)	60.0 ± 8.0
Body mass (kg)	93.2 ± 7.7
Height (cm)	177 ± 6.6
BMI (kg/m^2^)	29.9 ± 2.4
Waist circumference (cm)	101 ± 6.3
Hip circumference (cm)	109 ± 10.6
Waist to hip ratio	0.93 ± 0.07
Systolic blood pressure (mm Hg)	132 ± 11.7
Diastolic blood pressure (mm Hg)	80.2 ± 8.0
Peak flow (L/min)	498 ± 100
Smoking status	
Current smoker	0 (0)
Previous smoker	3 (13)
Ethnicity	
White British	16 (100)
Marital status	
Married	11 (69)
Single	3 (19)
Cohabiting	1 (6)
Divorced	1 (6)
Highest education	
High school	4 (25)
College	5 (31)
Undergraduate	0 (0)
Postgraduate	5 (31)
Doctorate	2 (13)
Employment status	
Employed full‐time	4 (25)
Employed part‐time	1 (6)
Self‐employed	4 (25)
Retired	7 (44)

### 3.1 Exercise bout

The median heart rate reserve during the aerobic intervals was 59.7% (IQR 57.6%‐61.8%), indicating high fidelity to the exercise protocol. The median RPE was 13.2 (IQR 12.7‐13.8) and power output was 93.8 W (IQR 81.7‐105 W). All participants completed all six aerobic intervals, apart from one participant who completed five intervals because of technical difficulties monitoring blood pressure, which is an indication for exercise termination.^28^

### 3.2 Acute exercise‐conditioned serum reduces LoVo cell proliferation

**FIGURE 1:**
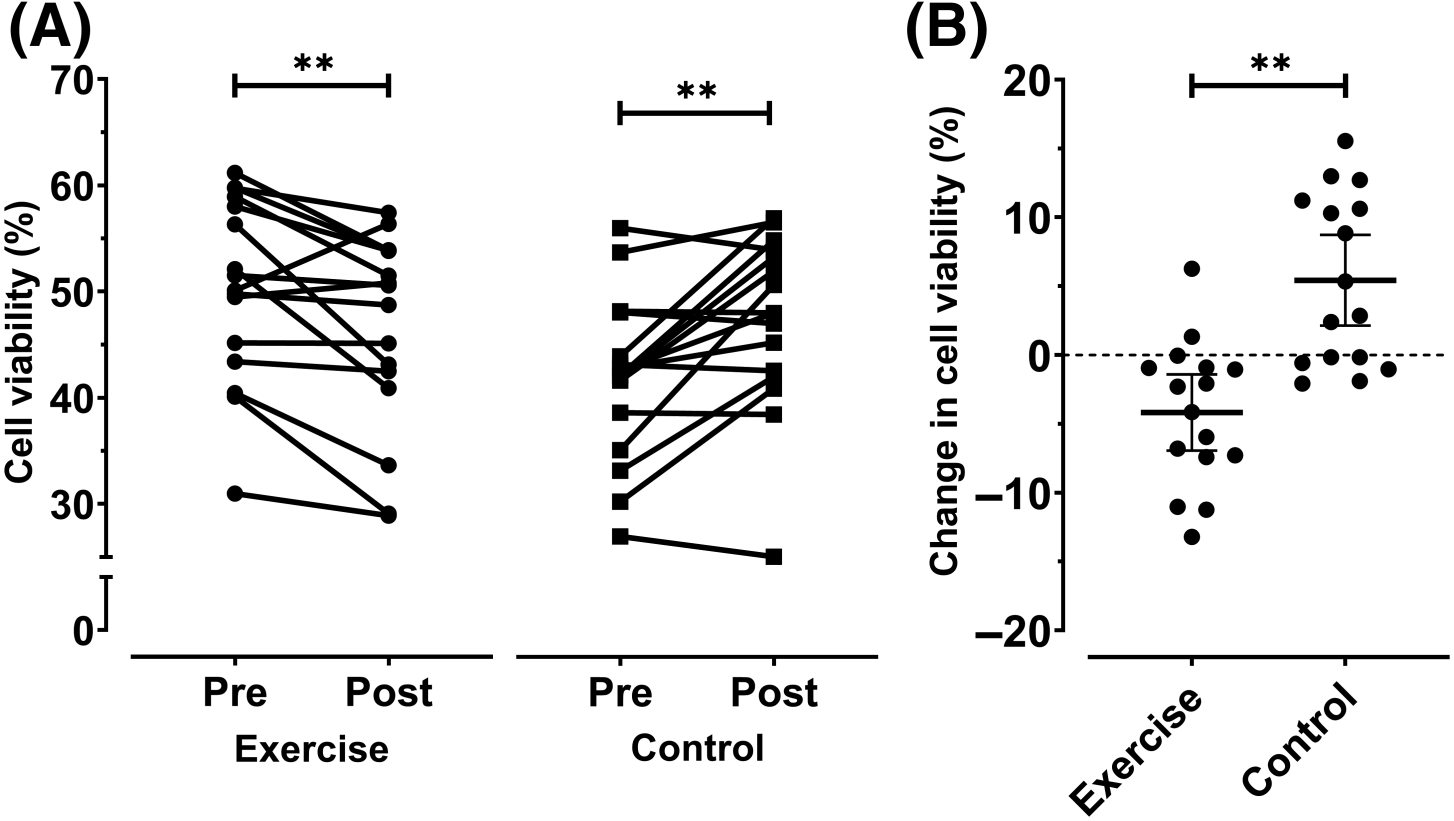
Effect of acute aerobic exercise‐conditioned serum on colon cancer cell growth. (A) LoVo cell viability following 48 hours of incubation with medium containing 10% human serum collected before and after an acute bout of aerobic exercise and a nonexercise control experiment. (B) Change in serum‐stimulated cell viability in the exercise and control experiments (mean ± 95% confidence interval). **P < .01

We incubated LoVo cells for 48 hours with medium containing 10% human serum collected before and after the acute exercise bout and a nonexercise control experiment. Stimulating LoVo cells with postexercise serum decreased cell proliferation compared to preexercise serum (−4.2%, 95% CI −6.8 to −1.5%; *P* = .006; Figure 1A), whereas postcontrol serum led to an increase in cell proliferation compared to precontrol serum (5.4%, 95% CI 2.2 to 8.6%; *P* = .003). After controlling for prevalues, exercise‐conditioned serum reduced cell proliferation compared to control (−5.7%, 95% CI −8.8 to −2.6%; *P* = .002; Figure 1B).

### 3.3 Acute exercise‐conditioned serum reduces intracellular γ‐H2AX expression

**FIGURE 2:**
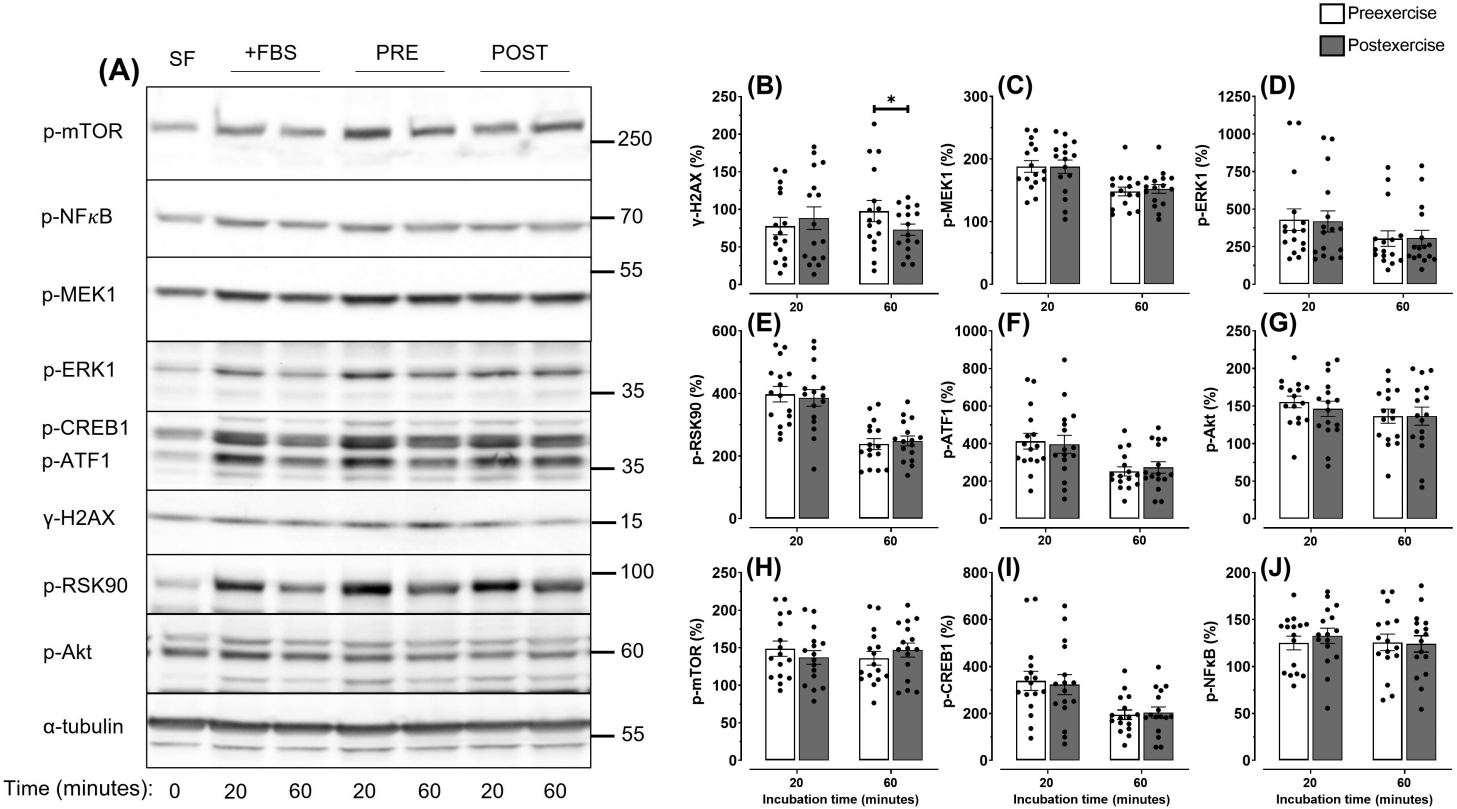
Effect of acute aerobic exercise‐conditioned serum on protein expression in LoVo cells. (A) Representative immunoblots of p‐MTOR, p‐NFκB, p‐MEK1, p‐ERK1, p‐CREB1, p‐ATF1, γ‐H2AX, p‐RSK90, p‐Akt and α‐tubulin in LoVo cells following 20 to 60 minutes of incubation with medium containing 10% human serum collected before and after an acute bout of aerobic exercise. (B‐J) Quantification of protein expression in LoVo cells (mean ± SEM). Bonferroni corrections were applied to adjust for multiple comparisons across the two time‐points (20 and 60 min). *P < .05. FBS, fetal bovine serum; POST, postexercise; PRE, preexercise; SF, serum free

To explore potential mechanistic pathways by which exercise suppressed colon cancer cell proliferation, we quantified the expression of various candidate proteins in LoVo cells after incubation with exercise‐conditioned serum for 20 to 60 minutes (Figure 2A). Exercise decreased levels of γ‐H2AX compared to preexercise serum after 60 minutes of incubation (−24.6%; *P* = .029; Figure 2B), indicating a reduction in DNA damage. In contrast, there was no evidence of an effect of exercise‐conditioned serum on p‐MEK1, p‐ERK1, p‐RSK90, p‐ATF1, p‐Akt, p‐mTOR, p‐CREB1 or p‐NFκB (all *P* > .05; Figure 2C‐J).

### 3.4 Acute aerobic exercise increases serum IL‐6

**FIGURE 3:**
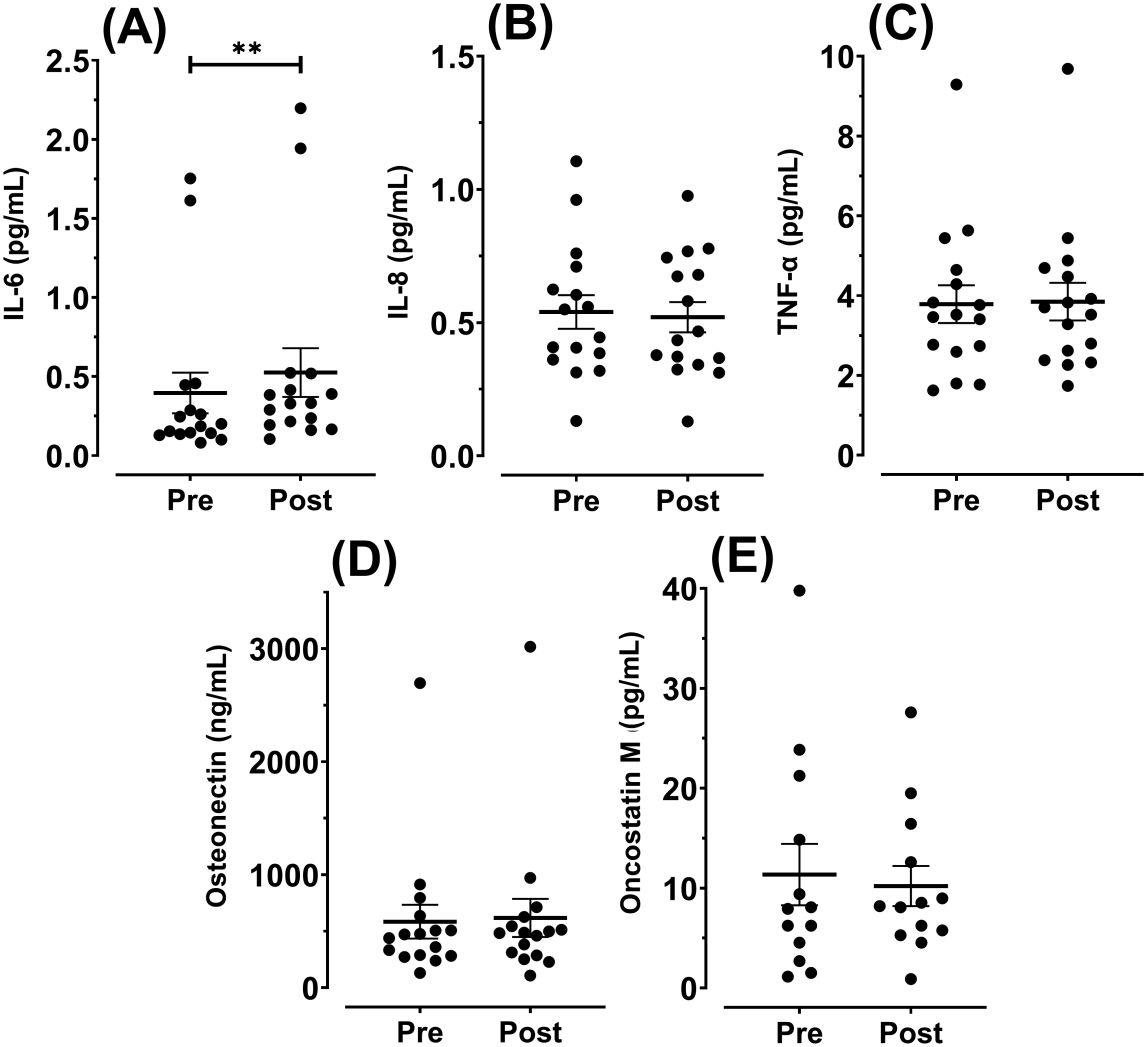
Effect of acute aerobic exercise on serum cytokine concentration. (A‐E) Concentrations of interleukin 6 (IL‐6), IL‐8, tumour necrosis factor‐alpha (TNF‐α), osteonectin and oncostatin M in pre‐ and postexercise serum (mean ± SEM). **P < .01

We then evaluated whether exercise modulated the serum concentration of seven cytokines to identify humoral factors that could be responsible for the exercise‐induced suppression of cell proliferation and DNA damage. Serum IL‐6 increased from pre‐ to postexercise (24.6%, 95% CI 11.2 to 37.9%; *P* = .002; Figure 3A), whereas there was no evidence of an effect of exercise on serum IL‐8, TNF‐α, osteonectin, or oncostatin M (all *P* > .05; Figure 3B‐E). IL‐10 and irisin were undetectable in the serum samples.

### 3.5 IL‐6 reduces LoVo cell proliferation and γ‐H2AX expression in a dose‐response manner

**FIGURE 4:**
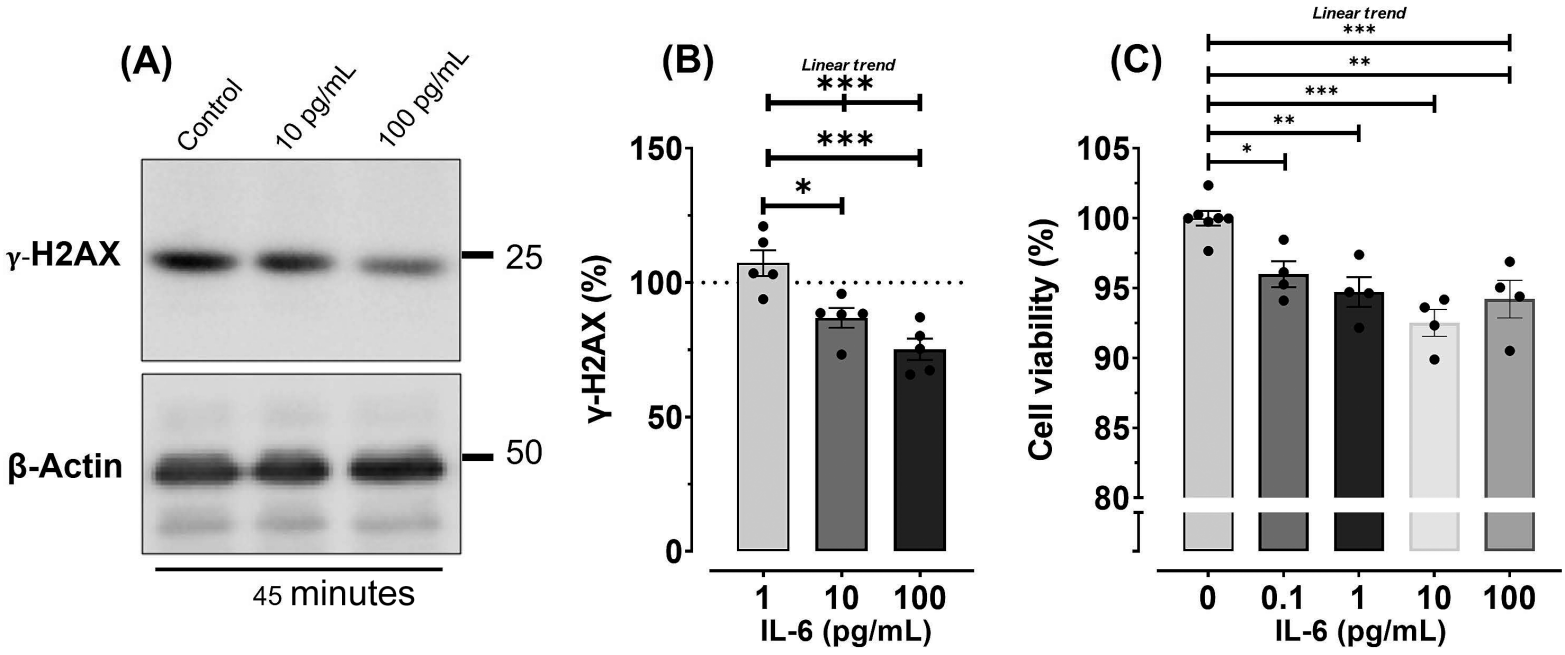
Effect of interleukin‐6 (IL‐6) on LoVo cell proliferation and intracellular γ‐H2AX expression. (A) Representative immunoblots of γ‐H2AX expression in LoVo cells after stimulation with actin and 0, 10 and 100 pg/mL of recombinant IL‐6 for 45 minutes. (B) Quantification of γ‐H2AX expression in LoVo cells exhibiting a dose‐response effect of IL‐6 (mean ± SEM of five repeat experiments). (C) Quantification of LoVo cell proliferation 48 hours after direct stimulation with recombinant IL‐6, showing a dose‐response effect (mean ± SEM of eight repeat experiments for control and four repeat experiments for IL‐6 doses). Bonferroni corrections were applied to adjust for multiple comparisons. *P < .05; **P < .01; ***P < .001

Given that acute exercise concomitantly increased serum IL‐6 and reduced LoVo cell proliferation and intracellular levels of γ‐H2AX, we proceeded to explore whether IL‐6 could directly regulate LoVo cell proliferation and γ‐H2AX. We stimulated LoVo cells with recombinant IL‐6 (HumanKine human IL‐6, Proteintech, UK) and demonstrated dose‐dependent effects (Figure 4A). Specifically, IL‐6 doses of 10 pg/mL (−20.4%, 95% CI −36.8 to −4.0%; *P* = .014) and 100 pg/mL (−32.1%, 95% CI −48.5 to −15.7%; *P* < .001) reduced γ‐H2AX expression compared to 1 pg/mL (Figure 4B). IL‐6 doses of 0.1 pg/mL (−4.0%, 95% CI −7.9 to −0.1%; *P* = .040), 1 pg/mL (−5.3%, 95% CI −9.2 to −1.4%; *P* = .004), 10 pg/mL (−7.5%, 95% CI −11.4 to −3.6%; *P* < .001), and 100 pg/mL (−5.8%, 95% CI −9.7 to −1.9%; *P* = .002) also reduced LoVo cell proliferation compared to control (Figure 4C). Furthermore, there was evidence of a linear trend for cell proliferation (*β* = −5.3%, 95% CI −7.4 to −3.2%; *P* < .001) and γ‐H2AX expression (*β* = −22.7%, 95% CI −31.6 to −13.8%; *P* < .001), demonstrating that as the IL‐6 dose increased, LoVo cell proliferation and γ‐H2AX levels decreased proportionately.

## 4 DISCUSSION

Our results show that acute aerobic exercise‐conditioned serum reduced colon cancer cell proliferation in vitro. This was accompanied by decreased levels of intracellular γ‐H2AX, indicating a reduction in DNA damage. Acute exercise also increased serum IL‐6, and stimulating colon cancer cells with recombinant IL‐6 reduced intracellular γ‐H2AX expression and cell proliferation in a dose‐dependent manner, mimicking the effect of exercise. Thus, our findings suggest that the inhibitory effects of exercise on colon cancer cell proliferation may be partly driven by IL‐6‐induced regulation of DNA damage and repair.

Epidemiological evidence suggests that regular physical activity reduces the relative risk of primary and recurrent colon cancer.^1^, ^3^, ^4^ However, the molecular mechanisms of action are poorly understood. Current dogma suggests physical activity reduces colon cancer risk through regulation of systemic insulin and proinflammatory cytokines,^5^, ^6^ but these effects seem to be tightly regulated by adiposity rather than physical activity per se.^29^, ^30^, ^31^ Here, we have demonstrated that stimulating colon cancer cells with serum obtained immediately after exercise reduces cell proliferation by ≈6%. This finding suggests that bioactive molecules released into the systemic circulation during exercise elicit biological effects on colon cancer cells to regulate cell proliferation. This proposed mechanism of action may partly explain the epidemiological associations between regular physical activity and reduced colon cancer risk.

Our findings align with studies using ex vivo colorectal tissue samples. For example, a 12‐month randomised controlled trial reported that aerobic exercise reduced the proliferative capacity of colon crypt cells in men who exercised at least 250 min·wk^−1^.^32^ A nonrandomised controlled trial in rectal cancer patients also showed that 6 weeks of preoperative exercise following neoadjuvant chemoradiotherapy led to greater tumour regression grading at the time of surgery,^33^ highlighting the potential of exercise to alter tumour morphology in vivo as well as in vitro*/*ex vivo.

γ‐H2AX is a sensitive biomarker for DNA double‐strand breaks (DSBs) and is formed following phosphorylation on the 139th serine residue of the histone variant H2AX.^34^ The oncogene‐induced DNA damage model for cancer development^23^ proposes that DNA DSBs drive the early stages of carcinogenesis. According to this model, aberrant cell proliferation prompted by activated oncogenes induces DNA replication stress.^23^ This replication stress results in the collapse of DNA replication forks, leading to the formation of DNA DSBs. LoVo cells harbour a mutation to the *KRAS* oncogene, and activated *RAS* has previously been shown to induce DNA DSBs in NIH3T3 fibroblasts within a single cell cycle.^35^ The sustained formation of DSBs contributes to genomic instability, which is a hallmark of cancer cells and increases the propensity of acquiring additional genetic mutations favouring cancer progression.^36^ Accordingly, the expression of γ‐H2AX is positively correlated with the malignant progression of human colorectal carcinoma^37^, ^38^ and with colon cancer cell line (HCT15) proliferation.^38^

We found that the exercise‐induced reduction in colon cancer proliferation was accompanied by decreased levels of intracellular γ‐H2AX, indicating a reduction in DNA damage. The phosphorylation status of major signalling molecules downstream of the *KRAS* oncogene were unaffected by exercise stimulation (Figure 2). In line with the oncogene‐induced DNA damage model for cancer development,^23^ this suggests that exercise may not have prevented the initial formation of DNA DSBs, although we cannot rule out that signalling pathways downstream of other oncogenes (eg, *APC*) were not altered by exercise. Instead, given that the disappearance of γ‐H2AX following DNA damage indicates repair of DSBs,^39^ it is possible that exercise facilitated DNA DSB repair. The capacity of exercise to enhance DNA repair has been highlighted previously,^40^ with potential mechanisms including increased free radical scavenger enzyme activity,^41^ increased DSB repair protein content such as Ku70,^42^ or attenuation of telomere attrition.^43^ Cancer cell lines are known to rapidly acquire new genetic variants in culture,^44^ and LoVo cells have the hypermutator phenotype microsatellite instability^21^ (a form of genomic instability caused by a defective DNA mismatch repair system). Therefore, the exercise‐induced repair of DNA DSBs may have shifted the cancer cells towards a more genetically stable phenotype,^45^ reducing the acquisition of further genetic mutations in culture and subsequently reducing cell proliferation.

Acute exercise increased serum IL‐6, which is consistent with evidence showing that IL‐6 is expressed and secreted by contracting skeletal muscle during exercise.^46^ We also found that directly stimulating LoVo cells with recombinant IL‐6 reduced γ‐H2AX expression and cell proliferation in a dose‐dependent manner, mimicking the effect of exercise. Taken together, the findings suggest that IL‐6 signalling may have driven exercise‐induced regulation of DNA damage. In support of this, direct IL‐6 treatment has been shown to reduce γ‐H2AX expression in oral squamous cell carcinoma cells^47^ and promote DNA repair in CD133‐positive cancer stem‐like cells after irradiation.^48^ IL‐6 has also been shown to cause cell cycle arrest, reduce proliferation, and activate DNA repair enzymes following partial hepatectomy in mice.^49^

IL‐6 is a pleiotropic cytokine and appears to play a dual role in cancer progression, which may depend on the duration of exposure. Lee and colleagues^50^ reported that acute exposure to IL‐6 (less than 28 passages) reduced prostate cancer cell growth, but long‐term exposure (more than 42 passages) increased cancer cell growth and IL‐6 mRNA expression. Thus, short‐term IL‐6 exposure may inhibit cancer growth through endocrine/paracrine signalling, while chronic exposure may lead to autocrine cell growth stimulation by inducing cells to acquire endogenous IL‐6 production.^50^ Acute exercise transiently increases circulating IL‐6 and prolonged exercise training reduces resting IL‐6 levels.^46^ Evidence of the dual role of IL‐6 indicates that these opposing systemic responses to acute and chronic exercise may both contribute to the inhibition of cancer progression.

While other serum markers were unaltered by exercise in our study, it is unlikely that the growth‐inhibitory effect of exercise was driven exclusively by IL‐6. Exercise elicits widespread effects on multiple organ systems and leads to the secretion of thousands of metabolites, peptides and RNA species.^14^ Thus, the preventive effect of acute exercise with respect to colon cancer is likely to result from a coordinated response of many diverse humoral factors and intracellular signalling pathways. Multiomic profiling of exercise‐conditioned serum and colon cancer cell lines following serum stimulation, combined with bioinformatics analytic tools, may help elucidate the complex molecular networks involved.

Our study has several strengths. In contrast to previous research,^18^ we incorporated a nonexercise control condition in a randomised crossover design to determine the effects of exercise‐conditioned serum on cancer cell proliferation. The inclusion of a control experiment accounts for biological variation and potential regression to the mean effects,^19^ thus providing a more accurate estimate of the true treatment effect. We also controlled for physical activity and dietary intake prior to the experimental sessions. Moreover, we prospectively registered the protocol and primary outcome and have made the data and code available on OSF,^25^ allowing researchers to computationally reproduce our results.

A limitation of our study is that the two‐dimensional cell culture model used does not fully reflect in vivo tumour morphology or microenvironment. Moreover, in contrast to the assay used in our study, bioactive molecules released during exercise in vivo must travel a potentially long distance in the systemic circulation to reach aberrant colonic epithelial cells. Ligands lacking a secretory signal sequence and those with a low molecular weight (and hence short residency time in serum) may be transported in small membranous extracellular vesicles (EVs).^14^ Further research is warranted to investigate whether plasma EVs mediate interorgan crosstalk during exercise by transporting bioactive molecules to distant aberrant cells. Furthermore, future research could use a colonic adenoma model to explore the effects of exercise on the growth of premalignant colorectal lesions, which is perhaps more relevant to cancer prevention than cancer cell‐based assays. A further study limitation is that most of the secondary outcomes were not prespecified, although all deviations from the preregistered protocol have been documented and justified (Table S1). Deviations included the measurement of some serum markers and the assessment of intracellular signalling pathways following stimulation with exercise serum.

To conclude, aerobic exercise‐conditioned serum reduced colon cancer cell proliferation in vitro, which appeared to be driven by IL‐6‐induced regulation of DNA damage and repair. This mechanism of action may at least partly underlie epidemiological associations linking regular physical activity with reduced colon cancer risk.

## CONFLICT OF INTEREST

The authors have no conflicts of interest to declare.

## AUTHOR CONTRIBUTIONS

Samuel T. Orange: Conceptualisation, Methodology, Validation, Formal Analysis, Investigation, Data Curation; Writing—Original Draft, Visualisation. Alistair R. Jordan: Methodology, Investigation, Resources, Writing—Review & Editing. Adam Odell: Methodology, Validation, Investigation, Resources, Writing—Review & Editing, Visualisation. Owen Kavanagh: Investigation, Resources, Writing—Review & Editing. Kirsty M. Hicks: Methodology, Investigation, Writing—Review & Editing. Tristan Eaglen: Investigation, Writing—Review & Editing. Stephen Todryk: Methodology, Resources, Writing—Review & Editing, Supervision. John M. Saxton: Conceptualisation, Methodology, Writing—Review & Editing, Supervision. The work reported in the paper has been performed by the authors, unless clearly specified in the text.

## ETHICS STATEMENT

The study was approved by the Faculty of Health and Life Sciences Ethics Committee at Northumbria University (ref: #17596) and informed consent was obtained from all participants before taking part in this research. The study was prospectively registered at ClinicalTrials.gov (ID: NCT04057274) and minor deviations from the original protocol are documented and justified in Table S1.

## Supporting information


**Appendix S1**: Supporting Information.

## Data Availability

All data and code are available on the Open Science Framework project page (https://osf.io/trw78/). Further information is available from the corresponding author upon request.
