# Supplementary material for: Acute aerobic exercise‐conditioned serum reduces colon cancer cell proliferation in vitro through interleukin‐6‐induced regulation of DNA damage
Source: Int J Cancer. 2022 Mar 5;151(2):265–74. doi: 10.1002/ijc.33982 (PMC9314683; doi:10.1002/ijc.33982)
Supplement: Supplementary file 1 — Appendix S1: Supporting Information. [file IJC-151-265-s001.pdf]

Corresponding author:
Dr Samuel T. Orange
School of Biomedical, Nutritional and Sport Sciences
Faculty of Medical Sciences
Newcastle University, Newcastle upon Tyne, NE2 4DR

Acute aerobic exercise-conditioned serum reduces colon cancer cell proliferation through IL-6-induced regulation of DNA damage *in vitro*

Samuel T. Orange, Alistair R. Jordan, Adam Odell, Owen Kavanagh, Kirsty M. Hicks, Tristan Eaglen, Stephen Todryk, John M. Saxton

Table of Contents

Supplementary figures 2
Supplementary tables 3

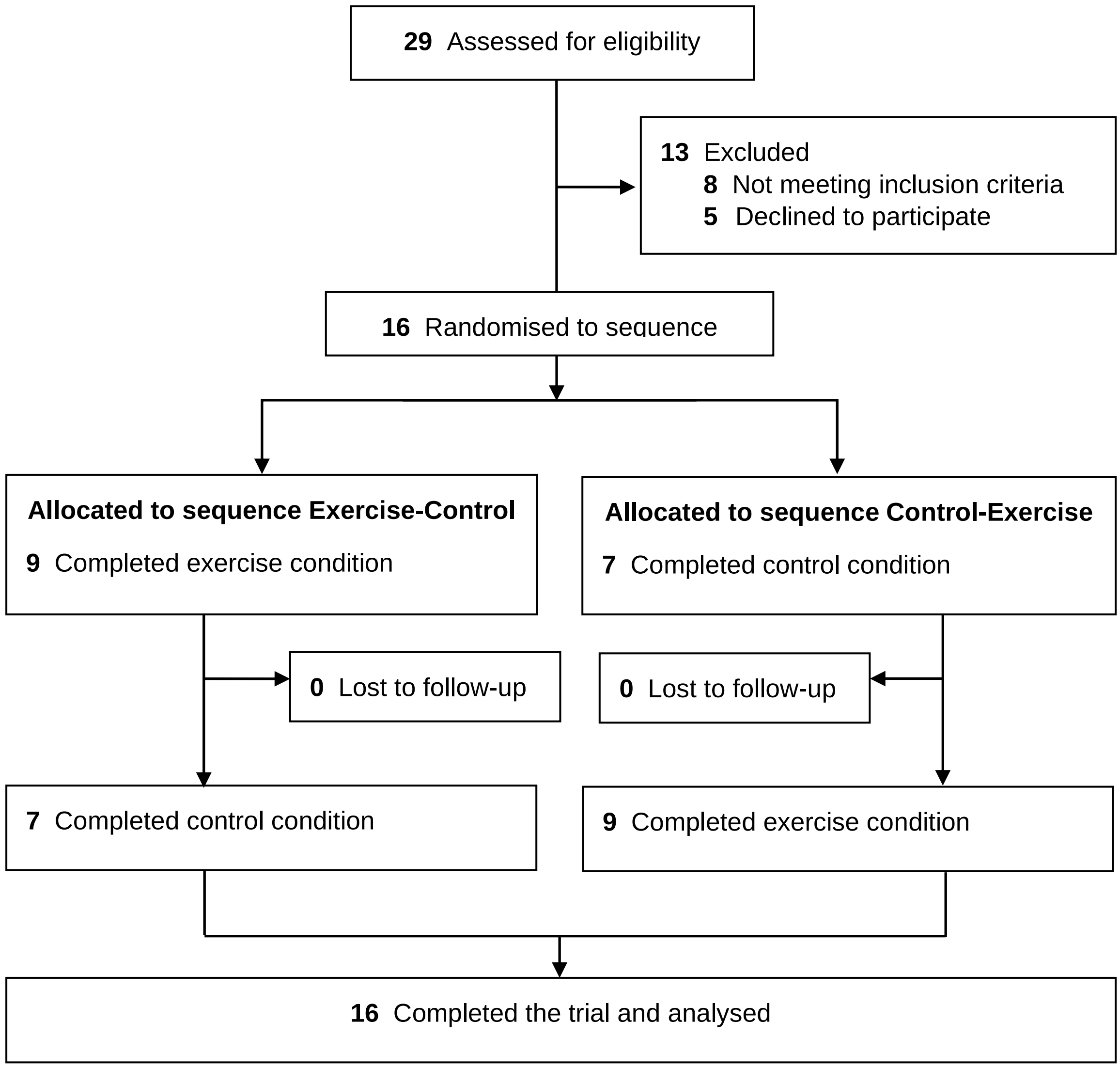

Supplementary Figure 1. Trial flow

Supplementary Table 1. Deviations to the prospectively registered protocol

	Deviation	Justification
#1	Measuring the effect of acute exercise on serum markers that pre-specified	We initially planned to measure serum insulin, IGF-1, epinephrine, norepinephrine, TNF-α, and IL-6. However, we decided to measure serum IL-6, IL-8, IL-10, irisin, osteonectin, and oncostatin M because of they have recently emerged as candidate cytokines linking exercise to cancer control.
#2	Evaluating the effect of exercise-conditioned serum and recombinant IL-6 on the expression of candidate proteins in LoVo cells	Our results showed that acute-exercise conditioned serum reduced colon cancer cell growth, and we wanted to explore potential molecular mechanisms underlying the effect. These experiments add important information to the results.

IGF-1 = insulin-like growth factor 1; IL-6 = interleukin-6; TNF-α = tumour necrosis factor-alpha
